# A *de novo TNNI3K* variant aggravates the pathogenicity of *DMD*-associated early-onset cardiomyopathy: a case report

**DOI:** 10.3389/fgene.2025.1525941

**Published:** 2025-03-11

**Authors:** Di Qie, Yang Zhai, Fan Yang, Yifei Li, Rong Xu

**Affiliations:** ^1^ Key Laboratory of Birth Defects and Related Diseases of Women and Children of MOE, West China Second University Hospital, Sichuan University, Chengdu, Sichuan, China; ^2^ Department of Pediatrics, West China Second University Hospital, Sichuan University, Chengdu, Sichuan, China; ^3^ Department of Emergency, Chengdu Women’s and Children’s Central Hospital, School of Medicine, University of Electronic Science and Technology of China, Chengdu, Sichuan, China; ^4^ Department of Radiology, West China Second University Hospital, Sichuan University, Chengdu, Sichuan, China

**Keywords:** *TNNI3K*, Duchenne muscular dystrophy, cardiomyopathy, myocardial fibrosis, magnetic resonance imaging

## Abstract

**Background:**

Dystrophin is a *DMD* coding protein that serves as a connector maintaining the structural formation and functional hemostasis of myofilaments, which regulate the contraction of cardiomyocytes. However, early-onset heart failure or cardiomyopathy is closely associated with adverse clinical outcomes in Duchenne muscular dystrophy (DMD)-affected patients. Pathogenicity screening and identification of the potential combined variants are thus critical for the management of such patients. Herein, we report a rare case of a patient with early-onset DMD attributed to a compound genetic variant in the *DMD* and *TNNI3K* genes.

**Case presentation:**

The proband, a 15-month-old male patient, presented with severe heart failure, enlarged ventricles, and diffuse fibrosis. Whole-exome sequencing was used to identify a compound missense variant as c.1540G>T (p.V514L) of the *DMD* gene and c.1633G>T of the *TNNI3K* gene, resulting in disease. The protein structures of the mutant dystrophin and TNNI3K were built using AlphaFold3. The amino acid residues around site 514 had changed in DMD p.V514L, and the altered surrounding structures resulted in protein dysfunction. Furthermore, the amino acid residues around site 545 had changed in TNNI3K p.G545C, causing significant alterations to the hydrogen bonding. As both of these mutations contribute to regulating the myofilaments, potential interactions are suspected. Then, the binding structure was established using AlphaFold3, and the structural changes were identified based on the compound variants.

**Conclusion:**

We present a rare case of a compound genetic variant that induces severe and very-early-onset heart failure in DMD patients. The compound variant attenuates the interactions between *DMD* and *TNNI3K*, leading to functional collapse of the myofilaments. This finding emphasizes the importance of comprehensive genetic analysis in DMD patients. Identification of additional variants can significantly aggravate the pathological process and disease prognosis, and such patients always require swift and careful clinical management to obtain desirable outcomes.

## 1 Introduction

Duchenne muscular dystrophy (DMD) is an X-linked recessive disorder caused by loss-of-function mutations in the *DMD* gene that encodes dystrophin; DMD manifests as progressive multisystem muscular degeneration with a predilection for skeletal, diaphragmatic, and cardiac tissues (Shirokova and Niggli, 2013). The molecular pathogenesis of DMD involves disrupted dystrophin–glycoprotein complex (DGC) formation, which compromises the structural integrity of the sarcolemma and precipitates dysregulation of the mechanotransduction pathway. This molecular instability allows pathological calcium influx through the stretch-activated channels (TRPC1/Piezo1) to initiate cascades of calpain-mediated proteolysis, mitochondrial permeability transition pore activation, and ultimately myocyte necrosis (Angulski et al., 2023; Gandhi et al., 2024). The cardiac sequelae progress through distinct phases, namely initial subclinical cardiomyocyte apoptosis (age 3–6 years), followed by compensatory interstitial fibrosis (TGF-β1-mediated), and eccentric hypertrophy, resulting in electrophysiological heterogeneity predisposing to ventricular arrhythmias and left-ventricular remodeling (De Giorgio et al., 2023). Longitudinal studies have demonstrated that this escalates the prevalence of cardiomyopathy from 25% at the age of 6 years to 59% by the age of 10 years, reaching near-universal penetrance (93%–100%) by 18 years; the phenotypic severity is strongly correlated with the *DMD* mutation type, where the frameshift variants in exons 45–50 (rod domain) confer delayed cardiac involvement compared to the C-terminal truncations that disrupt β-dystroglycan binding. This genotype–phenotype relationship underscores the dual role of dystrophin in structural stabilization and calcium homeostasis regulation (Shah and Yokota, 2023).

Emerging evidence suggests that the modifier genes governing cardiac pathophysiology can critically modulate the trajectory of DMD through protein interaction networks (Jefferies et al., 2005). Cardiomyopathy-associated variants are known to accelerate cardiac decompensation via two synergistic mechanisms, including hastening of left-ventricular remodeling through impaired calcium handling and destabilizing of the DGC–myofilament interface, that precipitate early-onset heart failure even in mild skeletal phenotypes. The cardiac-specific troponin-I-interacting kinase (TNNI3K) comprising N-terminal ankyrin repeats, a central serine/threonine kinase domain, and a C-terminal regulatory region orchestrates contractile regulation through phosphorylation of cardiac troponin I and modulation of β-adrenergic signaling (Pham et al., 2023). Genotype–phenotype correlation analyses have demonstrated that the *TNNI3K* variants alter myocardial stress responses and potentiate arrhythmogenic substrates. Although structural studies have confirmed that the actin-binding domain of dystrophin anchors the DGC to the myofilaments, emerging biophysical evidence suggests the existence of putative crosstalk between the hinge region of dystrophin and ankyrin domain of TNNI3K, indicating a novel mechanism by which dual dysfunction can exacerbate sarcomeric destabilization (Pham et al., 2024; Rieder et al., 2023). This pathophysiological framework underscores the need for combinatorial therapies targeting both membrane integrity and contractile regulation in the management of DMD-associated cardiomyopathy.

Herein, we report a rare case presenting with extremely early onset of heart failure as the initial clinical feature of DMD, which is attributed to a compound genetic variant in the *DMD* and *TNNI3K* genes. This case expands our understanding of the molecular mechanisms and functions of dystrophin in regulating myofilament contraction while emphasizing the importance of comprehensive genetic screening in DMD patients for improved clinical management and prognostic assessment.

## 2 Methods

### 2.1 Ethical considerations

This study was approved by the ethics committee of West China Second Hospital of Sichuan University (approval number: 2021–069). Written informed consent was obtained from the parents of the patients for whole-exome sequencing (WES) as well as inclusion of any clinical and imaging details in the publication.

### 2.2 Genetic testing

Peripheral blood samples were collected from the proband and his parents in ethylenediaminetetraacetic acid (EDTA) anticoagulant tubes and stored at 4°C for less than 6 h. DNA extraction was then performed using the Blood Genome Column Medium Extraction Kit (Tiangen Biotech, Beijing, China) according to manufacturer instructions.

### 2.3 WES and data analysis

WES was conducted using the NovaSeq 6000 platform (Illumina, San Diego, CA, United States), and the raw data were processed using FastP to remove any adapters and filter low-quality reads. The paired-end reads were aligned to the Ensembl GRCh38/hg38 reference genome using the Burrows–Wheeler aligner. Variant annotations were performed in accordance with database-sourced minor allele frequencies (MAFs) and practical guidelines on pathogenicity issued by the American College of Medical Genetics. The MAF annotations were based on the 1000 Genomes (1000G), dbSNP, ESP, ExAC, and Chigene inhouse MAF databases, while Provean, Sift, Polypen2_hdiv, and Polypen2_hvar databases were used for further annotations using R software (R Foundation for Statistical Computing, Vienna, Austria).

### 2.4 Protein structure analysis

The AlphaFold3 tool (https://golgi.sandbox.google.com/) was employed to establish the protein crystal structures and analyze the mutant sites of *DMD* and *TNNI3K*. Then, Pymol software was used to illustrate the molecular structures of the wild-type and mutant forms of the target genes. Additionally, AlphaFold3 was used to predict the potential interactions between dystrophin and TNNI3K proteins.

## 3 Clinical description and molecular results

### 3.1 History of illness and physical examination

A 15-month-old male patient presented to our emergency department with respiratory failure characterized by dyspnea, dysphonia, and non-productive cough. The clinical manifestations included perioral cyanosis upon exertion, lethargy, hyperhidrosis, reduced lacrimation, and peripheral coldness. The patient exhibited no pyrexia but developed hemoptysis with a pink frothy sputum. The proband, born at 38 weeks of gestation via caesarean section due to oligohydramnios, was the first offspring of non-consanguineous parents and had a birth weight of 2,850 g. The prenatal ultrasonography had revealed renal cystic changes, and the neonatal period was complicated by pneumonia that required a week-long period of hospitalization, during which ultrasonographic examination confirmed autosomal dominant polycystic kidney disease with concomitant left hydronephrosis. The patient’s medical history included one episode of urinary tract infection. The developmental history revealed feeding difficulties since birth, dental eruption at 10 months, and current inability to achieve independent standing. Language development was limited to maternal recognition without further vocabulary acquisition. Physical examination revealed distinctive craniofacial features, including a high-arched palate, hypertelorism, and low-set ears with thickened pinnae. Marked perioral cyanosis was evident during crying, which was accompanied by hoarse vocalization. Respiratory assessment demonstrated soft cervical tissues without tracheal tug, absent tripod positioning, and decreased left-lung breath sounds with prominent coarse crackles predominantly in the left field. Cardiac examination revealed irregular rhythm with diminished heart sounds and significant murmurs across all valve areas. The abdomen was soft with mild distention and hepatomegaly extending 3 cm below the costal margin; the spleen was non-palpable. Peripheral examinations showed no significant pitting edema or digital clubbing, along with preserved peripheral perfusion and normal digital temperature.

### 3.2 Laboratory and imaging evaluations

Laboratory investigations revealed electrolyte disturbances characterized by hyponatremia (Na^+^: 130.7 mmol/L), elevated creatine kinase-MB (7.43 μg/L), increased β-hydroxybutyrate (0.94 mmol/L), and markedly elevated B-type natriuretic peptides (>5,000.00 pg/mL), with the cTnI assessment demonstrating normal levels. Metabolomic analysis of the blood demonstrated elevated medium- and long-chain acylcarnitines (C14, C16:1-OH, C18, C18:1, and C18:2), suggesting underlying fatty-acid oxidation dysfunction. Urinary organic acid analysis revealed decreased excretion of 2-ketoglutarate-OX-2 (1) and homovanillic acid-2. Notably, comprehensive hematological, hepatic, renal, and thyroid function tests showed that the results were within the reference ranges. Additional parameters, including pyruvate, lactate, and ammonia concentrations, along with urinalysis and fecal examination showed no significant abnormalities. Autoantibody screening yielded negative results. Thoracic, cervical, and cerebral computed tomography revealed bilateral pulmonary inflammation with interstitial changes, marked cardiomegaly with consequent left main bronchus compression, minimal right-sided pleural effusion, and mild bilateral ventricular dilatation. Some additional findings included right submandibular gland enlargement, pharyngeal wall thickening, maxillary sinus mucosal hyperplasia, and bilateral cervical lymphadenopathy. Renal imaging demonstrated superior pole enlargement with irregular morphology. Renal ultrasonography confirmed bilateral nephromegaly with parenchymal echogenic changes, multiple cystic formations consistent with infantile polycystic kidney disease, left hydronephrosis, and partial right-sided collecting system dilatation. The abdominal ultrasonography was unremarkable.

**FIGURE 1:**
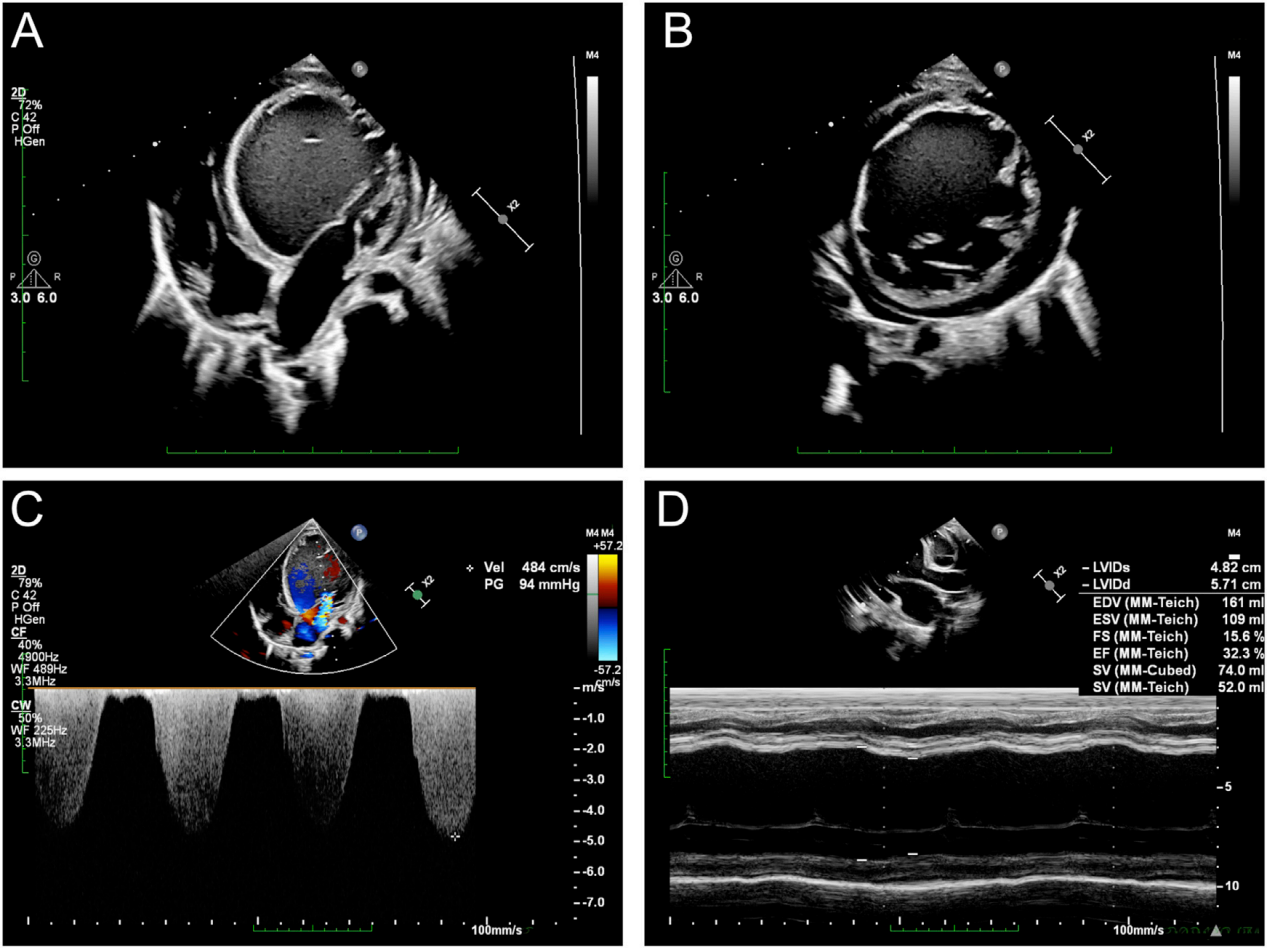
Echocardiographic assessments of the proband: **(A)** cardiac ultrasound demonstrates significantly enlarged left ventricle; **(B)** short-axis image reveals a hypertrabecularization in the left ventricle; **(C)** severe regurgitation observed at the mitral valve; **(D)** severe heart failure recorded by M-mode ultrasound.

Echocardiography revealed severe left-ventricular dysfunction, left-ventricular enlargement with altered echogenicity, severe mitral regurgitation, minimal pericardial effusion, and bilateral ventricular systolic dysfunction (Figure 1). Electrocardiography revealed sinus arrhythmia with junctional escape rhythm, axis deviation, left-ventricular hypertrophy, and T-wave abnormalities in leads II, III, aVF, V3, and V5. Twenty-four-hour Holter monitoring showed sinus arrhythmia, isolated atrial ectopy (8 events in 24 h), ventricular ectopy (28 events in 24 h, <0.1% of total beats) with paired morphologies, T-wave changes, and moderately reduced heart-rate variability (SDNN: 80 m).

**FIGURE 2:**
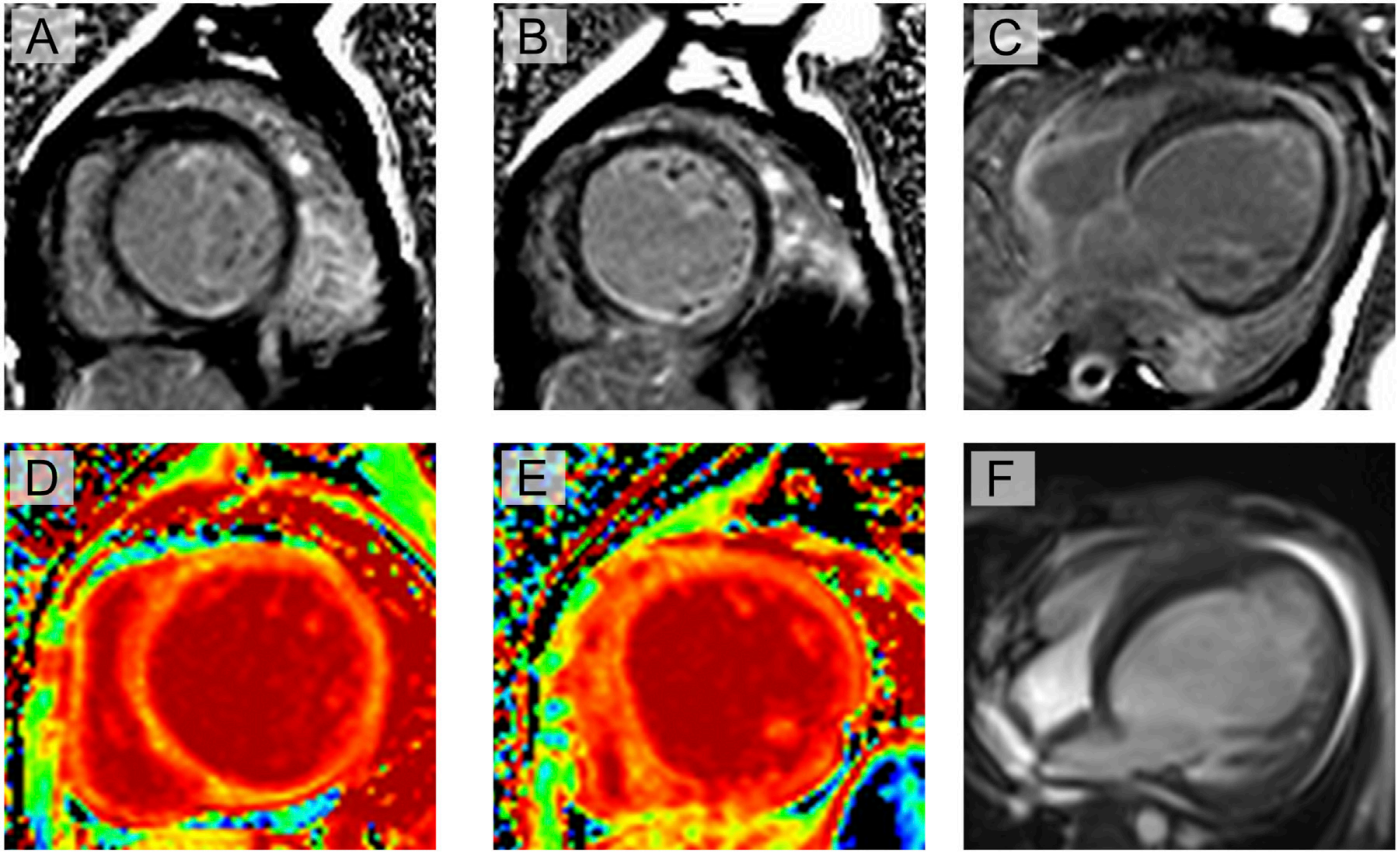
Cardiac magnetic resonance imaging evaluation of the proband: **(A–C)** late gadolinium enhancement shows myocardial fibrosis at multiple locations, especially in the muscle layer of the interventricular septal wall and subendocardial layer of the left ventricle (yellow arrow); **(D, E)** T1 mapping shows significantly increased extracellular volume; **(F)** cine imaging shows enlarged left ventricle, significantly reduced left-ventricular motion, and reduced systolic function (ejection fraction = 21%). Hypertrabecularization was also observed in the free wall and apex of the left ventricle.

Interestingly, cardiac magnetic resonance imaging demonstrated a different presentation compared to that observed in general DMD patients (Figure 2). The cardiac cine sequence showed that the left ventricle was enlarged, with significantly reduced left-ventricular motion and systolic function (ejection fraction (EF) = 21%). Hypertrabecularization was observed in the free wall and apex of the left ventricle. Late gadolinium enhancement and T1 mapping showed diffuse myocardial fibrosis, especially in the muscle layer of the interventricular septal wall and subendocardial layer of the left ventricle.

### 3.3 Molecular results

**FIGURE 3:**
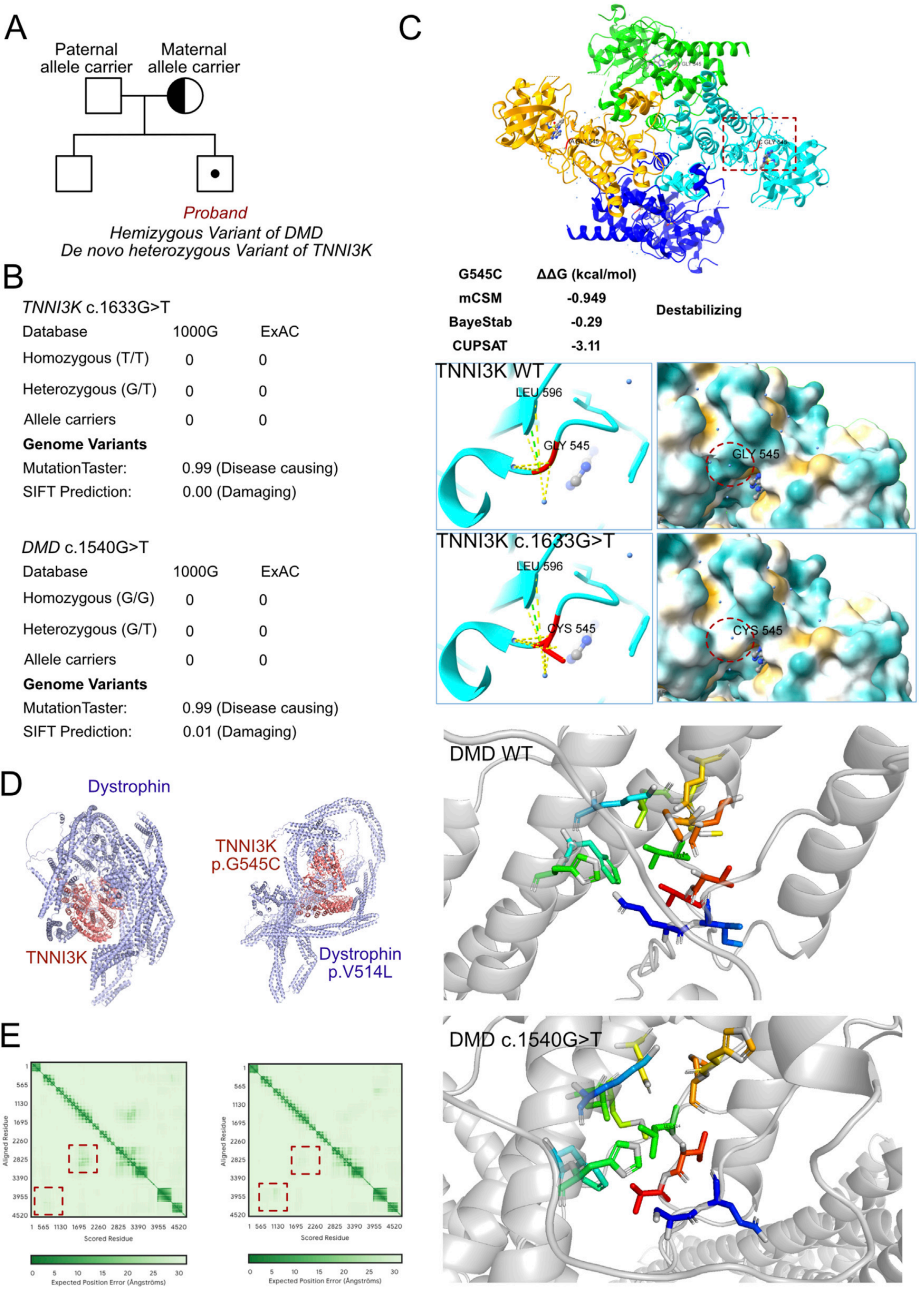
Molecular features of *TNNI3K* and dystrophin. **(A)** The proband exhibited hemizygous variants of *DMD* (c.1540G>T, p.V514L) and *TNNI3K* (c.1633G>T, p.G545C). **(B)** The variants of *DMD* (c.1540G>T) and *TNNI3K* (c.1633G>T) have never been reported in the ExAC and 1000G databases, and both were predicted for protein damage by MutationTaster and SFIT. **(C)** Protein structures of *TNNI3K* and dystrophin built using AlphaFold3 based on sequencing of the wild-type and mutant genes; the relationships between the residues around the mutant sites indicate structural changes due to the *DMD* p.V514L and *TNNI3K* p.G545C variants. **(D)** Potential interactions between *TNNI3K* and dystrophin molecules based on the wild-type and mutant amino acid sequences. **(E)** Prediction scores of the interacting residues within the established models between wild-type and mutant molecules of *TNNI3K* and dystrophin.

As unexplained severe heart failure was identified and a rare cardiomyopathy was suspected, genetic testing was conducted to explore any associated variants. WES was performed for the proband and their parents. According to the results of WES, a hemizygous variant was identified as c.1540G>T (p.V514L) in the *DMD* gene (Figure 3A); this allele variant was inherited from the maternal side, and the *DMD* c.1540G>T variant had never been reported in the ExAC and 1000G databases. Further potential compound variants were searched based on the WES results. In detail, we identified all potential cardiomyopathy-associated variants and validated the pathogenic or likely pathogenic variants. Then, careful screening was completed based on the MAFs, CADD scores, and potential correlations with the clinical manifestations of the proband. Next, a heterozygous variant was also identified as c.1633G>T (p.G545C) in the *TNNI3K* gene, which was absent in both the paternal and maternal sides and was treated as a *de novo* variant; this allele variant has also never been reported, which makes it a novel genotype (Figure 3B). Moreover, we excluded other potential variants involved in cardiovascular, metabolic, and muscular disorders. Then, we excluded all variants that were reported as pathogenic or likely pathogenic, and none of these were confirmed to be associated with the phenotype of the proband as heart failure and cardiomyopathy. Hence, we suspected that the compound variants of *DMD* c.1540G>T and *TNNI3K* c.1633G>T contributed to the pathogenic phenotype of this proband as a complicated manifestation of clinical cardiac dysfunction. To elucidate the molecular structures of the human *DMD* and *TNNI3K* genes, MutationTaster was used with R software to predict the pathogenicities of *DMD* c.1540G>T and *TNNI3K* c.1633G>T as well as assess the impacts of these two mutations on specific protein structures. Then, AlphaFold3 tool was used to establish the protein crystal structure and analyze the mutant sites of *DMD* and *TNNI3K* (Figure 3C) (Varadi et al., 2022; Jumper et al., 2021). Modeling analysis with proteins was performed using AlphaFold3 (https://golgi.sandbox.google.com/) for the mutant sites in the wild-type genes using Pymol software. We illustrate the molecular structural differences between the wild-type and mutant varieties of *DMD* and *TNNI3K*. The amino acid residues around site 514 had changed in DMD p.V514L, with alterations in the surrounding residues without labeling of the corresponding hydrogen bonds (Figure 3C), which coincided with the pathogenic predictions (Figure 3C). The relationship between the residues around the mutant site indicated that structural change owing to the variant of DMD p.V514L would cause protein dysfunction. The amino acid residues around site 545 had changed in *TNNI3K* p.G545C. According to the analysis results using ChimeraX software, after G545C substitution, the hydrogen bond network of the TNNI3K protein (green dashed line) did not change but the contact points with the water molecules (yellow dashed line) increased significantly; at the same time, the hydrophobic distribution of the protein showed that the hydrophobic region (orange yellow) increased and hydrophilic region (blue) decreased. In addition, free-energy difference analysis predicted that this substitution could cause decreased protein stability (ΔΔG<0). G545 is located in the serine/threonine kinase domain of *TNNI3K* and is a critical region for its function. The G545C substitution introduces the sulfhydryl group of cysteine, which increases the hydrophobicity of this site and could enhance its interactions with the surrounding hydrophobic groups, thereby affecting the overall conformation and stability of the protein. Such conformational changes may lead to alterations in the kinase activity or substrate binding capacity, which can in turn interfere with the normal function of *TNNI3K* (Figure 3C). Furthermore, we used AlphaFold3 to predict the potential interactions between *DMD* and *TNNI3k* (Figure 3D), and the results demonstrated that these genes could interact with each other at specific residues, where the C terminals of *TNNI3K* could come into contact with the N terminals of *DMD* (Figure 3E). However, the compound variants of *DMD* p.V514L and *TNNI3K* p.G545C can lead to significant changes in the structural predictions between these two proteins, implying that the original interactions between *DMD* and *TNNI3K* can collapse and the space folds of *DMD* itself may also be disrupted. Thus, the illustrations demonstrate that these single mutations definitely impair protein formation. Additionally, the combined mutations can aggravate molecular function collapse as these two proteins serve as a complex in maintaining myofilament hemostasis and biological functions to regulate cardiomyocyte contractions.

### 3.4 Final diagnosis and treatment

Upon analyzing the clinical manifestations as well as conducting imaging assessments and genetic tests, the patient was diagnosed with DMD accompanied by early-onset heart failure. Then, a long-term follow-up and therapy plan was suggested, including Sacubitril Valsartan Digoxin (0.15 mg daily), sodium (15 mg twice a day), Empagliflozin (2.5 mg daily), metoprolol (25 mg daily), spironolactone, and antiepileptic medication administration. Following therapeutic intervention, the patient demonstrated partial clinical improvement characterized by enhanced exercise tolerance and mild tachypnea within the compensatory physiological limits, accompanied by strengthened myocardial contractility with decreased arrhythmic events. Objective measurements revealed progressive reduction of hepatomegaly to 1 cm subcostal, consistent with diminished hepatic congestion. Although these parameters indicate positive therapeutic response, the residual symptomatology persists, necessitating ongoing cardiovascular monitoring and serial hepatic assessments to evaluate disease progression and treatment efficacy. Thus, the patient’s heart failure has been evaluated as irreversible, and a suggestion of heart transplantation has been provided to the patient, who is currently on the wait list.

## 4 Discussion

This case report highlights the complex interplay between genetic variants in the *DMD* and *TNNI3K* genes, resulting in extremely early onset of cardiomyopathy in a patient with DMD. This finding contributes to the growing body of evidence suggesting that multiple genetic variants can significantly influence the severity and progression of cardiac involvement in DMD.

Early-onset cardiomyopathy in infants presents unique challenges characterized by severe clinical manifestations and poor prognosis. In children under 2 years of age, the symptoms typically include failure to thrive, feeding difficulties, and respiratory distress, which often progresses rapidly to heart failure (Wilkinson et al., 2012). The genetic landscape of early-onset cardiomyopathy is diverse, encompassing mutations in the sarcomeric genes, mitochondrial DNA, and metabolic pathways, with certain mutations (e.g., calmodulin and RASopathy-associated) being linked to particularly severe presentations (Cahill et al., 2013; Norrish et al., 2019). Dilated cardiomyopathy has emerged as the predominant form in infants and is associated with severe systolic dysfunction as well as higher mortality rates (Lipshultz et al., 2019). The poor prognostic indicators include presentation before 12 months of age, presence of multiple genetic variants, severe systolic dysfunction at diagnosis, and requirement of mechanical circulatory support (Pagano et al., 2023; Wanert et al., 2023). The molecular pathogenesis of DMD-related cardiomyopathy entails multiple interconnected mechanisms stemming from dystrophin deficiency (Shirokova and Niggli, 2013). The absence of dystrophin disrupts the DGC, leading to mechanical fragility of the sarcolemma and impaired force transmission. This triggers a cascade of pathological events, including increased calcium influx, elevated oxidative stress, mitochondrial dysfunction, and activation of proteases, ultimately resulting in cardiomyocyte death and replacement fibrosis. The loss of dystrophin also affects the mechanotransduction and cellular signaling pathways, leading to altered nitric oxide synthase (NOS) signaling and impaired vascular functions. These mechanisms create a self-perpetuating cycle of cardiac damage that manifests as progressive cardiomyopathy in DMD patients (Kamdar and Garry, 2016).

Recent studies have demonstrated that multiple genetic variants can exacerbate the clinical manifestations and worsen prognosis in cardiomyopathy associated with DMD (Parvatiyar et al., 2019; Rubi et al., 2018; Mazzarotto et al., 2020). Some notable examples include combinations of DMD variants with titin (TTN) truncating variants, which typically present with early-onset muscle weakness and severe cardiomyopathy. Cases involving DMD and lamin A/C (LMNA) variants show particularly severe cardiac conduction defects and early-onset arrhythmias (Brayson and Shanahan, 2017). Similarly, patients with combined *DMD* and myosin heavy chain 7 (*MYH7*) variants demonstrate unique mixed-cardiac phenotypes, showing features of both dilated and hypertrophic cardiomyopathy. These dual-variant cases consistently show early onset of cardiac symptoms, more severe cardiac manifestations, and often require modified treatment approaches compared to typical DMD cases (Mazzarotto et al., 2020; El Refaey et al., 2017; Alsoufi et al., 2016).

In our patient, we identified a combined genetic variant in the *DMD* and *TNNI3K* genes. TNNI3K is a cardiac-specific MAPKKK that serves as a crucial regulator of heart function through multiple molecular mechanisms and genetic pathways (Wang et al., 2013). At the molecular level, *TNNI3K* modulates cardiac contractility via troponin I phosphorylation and influences calcium homeostasis while participating in cellular stress response pathways and metabolic regulation. The pathogenic variants of *TNNI3K* have been associated with a spectrum of cardiac conditions, including dilated cardiomyopathy, atrial fibrillation, and conduction system diseases (Gan et al., 2019; Theis et al., 2014).

Although direct physical interactions between dystrophin and *TNNI3K* have not been established strongly, the proteins participate in interconnected cellular networks that significantly influence cardiac functions. Their functional integration occurs through overlapping signaling pathways, where TNNI3K influences the MAP kinase cascades and dystrophin’s structural role affects mechanical-stress-induced signaling, both of which ultimately impact calcium handling and contractile functions in cardiomyocytes (Gan et al., 2019; Theis et al., 2014). Using AlphaFold3, we illustrated the potential interaction domains and protein structures between dystrophin and *TNNI3K*. In the wild-type gene sequences, *TNNI3K* showed a higher likelihood of interaction with *DMD*. However, in the mutant protein predictions, the crystal protein structure was significantly disrupted and potential binding sites were altered. This computational analysis supports our hypothesis of a cooperative regulation between *DMD* and *TNNI3K* in maintaining the basic and physiological functions of cardiomyocytes, particularly those involving cellular signaling and protein interactions.

## 5 Conclusion

Our case demonstrates that specific genetic variants can induce severe and very-early-onset cardiomyopathy in DMD patients. The interactions between the *DMD* and *TNNI3K* gene variants likely contribute to the extreme phenotype observed in our patient. These findings emphasize the importance of comprehensive genetic testing in DMD patients, particularly when unusual or severe cardiac manifestations are present. Identification of additional variants can also significantly influence patient management and outcomes, highlighting the need for personalized approaches in the care of DMD patients with cardiac involvement.

## Data Availability

The datasets presented in this study can be found in online repositories. The names of the repositories and accession numbers can be found in the article/supplementary material.
